# Magnesium Sulfate and Its Versatility in Anesthesia: A Comprehensive Review

**DOI:** 10.7759/cureus.56348

**Published:** 2024-03-17

**Authors:** Janhavi S Dahake, Neeta Verma, Dushyant Bawiskar

**Affiliations:** 1 Anesthesia, Jawaharlal Nehru Medical College, Datta Meghe Institute of Higher Education and Research, Wardha, IND; 2 Sports Physiotherapy, Abhinav Bindra Targeting Performance, Bengaluru, IND

**Keywords:** pain relief, postoperative, preoperative, epsom salt, anesthesia, mgso4

## Abstract

In the field of general anesthesia, magnesium sulfate (MgSO4) has become a valuable adjunct because it provides a range of benefits that enhance and optimize conventional aesthetic procedures. This review highlights the various intra-anesthetic benefits of MgSO4 while examining its complex function in the treatment using anesthesia. Magnesium inhibits the release of acetylcholine at the motor endplate and blocks calcium channels at presynaptic nerve terminals. This reduces the amplitude of endplate potential and the excitability of muscle fibers, which increases the potency of a neuromuscular blockade by nondepolarizing neuromuscular blockers. This activity may lessen the need for primary muscle relaxants. Moreover, its capacity to potentially reduce the total amount of main aesthetic agents needed emphasizes its function in maximizing anesthesia dosage, ensuring sufficient depth while perhaps potentially reducing adverse effects linked with increased dosages. MgSO4’s adaptable qualities present a viable path for improving anesthetic outcomes, possibly improving patient safety and improving surgical results.

## Introduction and background

Magnesium sulfate (MgSO4), a substance called Epsom salt, has become significant in several medical fields because of its many uses and therapeutic benefits [1]. This introduction aims to provide an overview of MgSO4's various functions and uses in anesthesiology. It is used medicinally in obstetrics, neurology, cardiology, and anesthesiology. The compound's ability to alter several biochemical pathways and cellular activities within the human body is the source of its physiological actions and therapeutic effects [2]. The care of preeclampsia and eclampsia during pregnancy is one of the most well-known uses of MgSO4. It is essential for controlling hypertension and preventing seizures in these dangerous situations because of its anticonvulsant solid properties and capacity to produce vasodilatory effects, which significantly lower the dangers to both the mother and the fetus [3]. MgSO4 is helpful in neurological conditions outside of pregnancy. Its neuroprotective qualities have been emphasized by studies [4], indicating that it may be used in other clinical contexts to treat disorders like stroke, traumatic brain damage, and seizures. Because of its broncho-dilatory properties, MgSO4 has also been studied for its potential to help reduce acute asthma exacerbations. MgSO4 is used in cardiology to treat some cardiac arrhythmias and as an additional therapy for certain cardiac diseases because of its vasodilatory qualities. At the same time, research on its exact function is still underway [5,6]. MgSO4 has additionally shown value in anesthesia. As an adjuvant to general anesthesia, it facilitates the relaxation of muscles, lowers the need for drugs such as propofol rocuronium, and may improve pain management following surgery. The administration of MgSO4, however, needs to be carefully considered and monitored due to its potential for adverse consequences, especially in situations of overdose or in patients with impaired renal function, despite its extensive use and variety of therapeutic applications [7,8].

MgSO4 has drawn a lot of interest in the field of general anesthesia. Although not the main aesthetic, its supplemental use in anesthesia protocols has demonstrated the potential to improve perioperative care and patient outcomes. Achieving the best possible muscle relaxation, managing discomfort like nausea and vomiting, and maintaining hemodynamic stability are essential components of aesthetic care for successful surgical procedures. Due to its well-known muscle relaxant, analgesic, and vasodilatory qualities, MgSO4 has become a valuable adjunct to supplement these essential aspects of aesthetic care [9,10]. This discussion intends to highlight the expanding importance of MgSO4 as an additional tool in enhancing aesthetic regimens by clarifying its mechanisms of action and clinical consequences. During surgeries, maintaining patient safety and surgical accuracy requires relaxed muscles. By competitively inhibiting calcium channels at the neuromuscular junction, MgSO4 is a muscle relaxant that helps achieve and sustain targeted degrees of muscle relaxation [11]. This property benefits surgical procedures that need immobility and precise control [12,13].

Furthermore, its analgesic qualities enhance those of conventional anesthetics, perhaps lowering the dosage of additional analgesics and minimizing their adverse effects. Due to its simultaneous effects on analgesia and muscular relaxation, MgSO4 is considered a significant adjuvant in achieving balanced anesthesia and efficient pain management. MgSO4 impacts firm tone and pain perception, but its vasodilatory properties [14] also help preserve hemodynamic stability when under anesthesia. Because of its capacity to dilate blood vessels, blood pressure may be maintained, and perfusion can be enhanced [15-17].

## Review

Methodology

**Figure 1:**
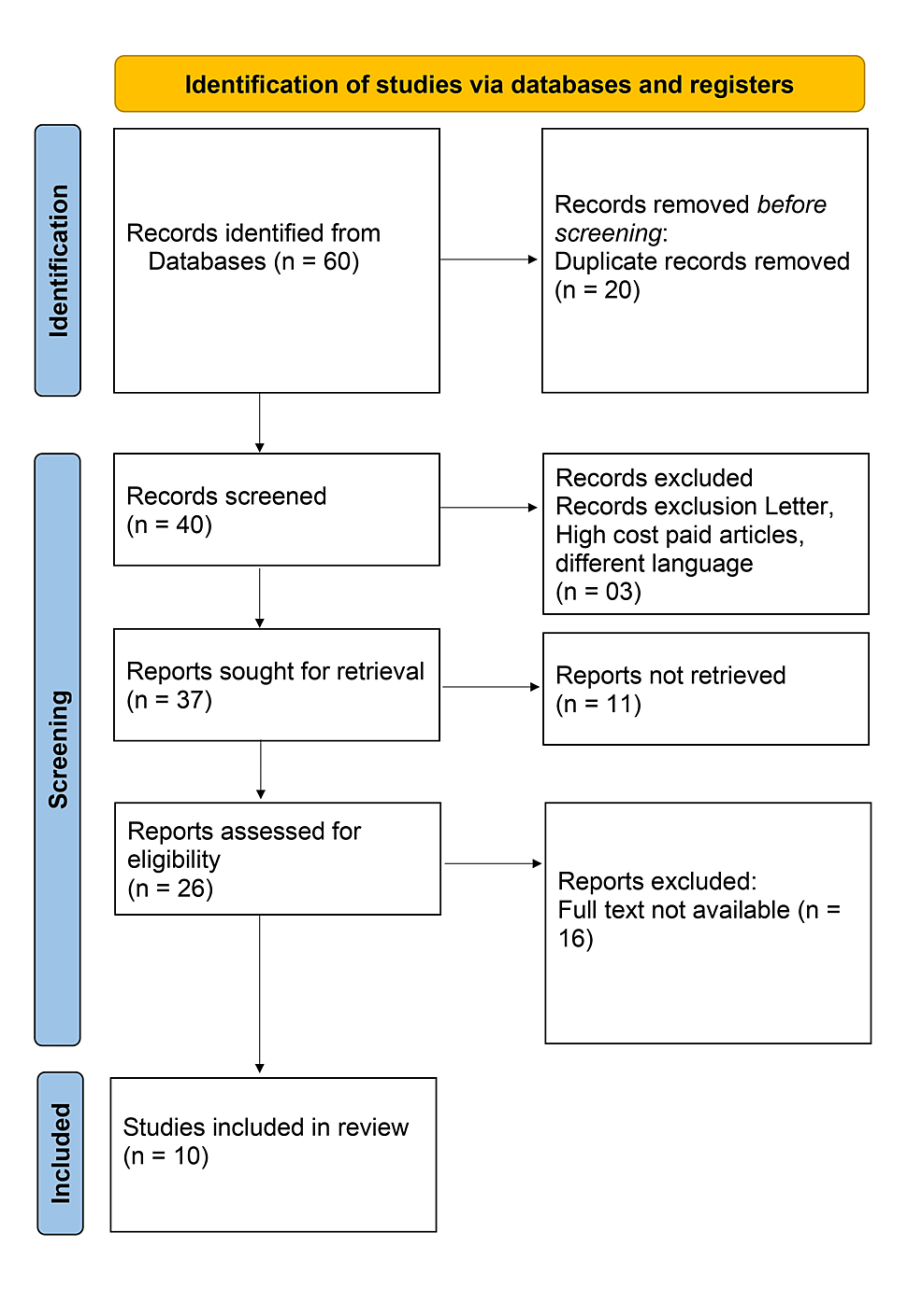
Prisma flow chart of list of included studies in the review

The methodology involves a comprehensive literature search strategy using multiple electronic databases, including PubMed, Scopus, and Google Scholar. The search terms used were related to “Magnesium sulfate”, “Anesthesia”, “Epsom salt”, and “Pain relief”. In addition to electronic database searches, the reference lists of relevant articles and review papers were manually searched for additional studies. Language restrictions were applied; only studies published in English and up to the current knowledge as of 2023 were included. The inclusion criteria were defined to select studies that were relevant and of high quality. Studies investigating the role of MgSO4 during anesthesia were included. This encompassed in vitro studies, animal models, and clinical studies of various designs. Exclusion criteria included studies focusing solely on MgSO4 or anesthesia and review articles, editorials, commentaries, and conference abstracts. Two independent reviewers screened the title and abstracts, followed by a full-text assessment of selected articles. Disagreements were resolved through consensus or consultation with a consensus if needed. The methodology aimed to include high-quality studies that contributed to the comprehensive understanding of the role of MgSO4 during anesthesia. By employing a rigorous search strategy and applying strict inclusion and exclusion criteria, a robust selection of studies was identified to inform the review (Figure 1, Table 1).

**Table 1:** List of studies included in the review

Author	Year	Type	Conclusion
Rajabi et al. [18]	2020	Research article	MgSO4 or clonidine infused intravenously would maintain hemodynamic parameters and deepen general anaesthesia in the context of surgical delivery, posing no additional acute danger to newborns.
Garcia et al. [19]	2021	Research article	Even in the presence of MgSO4, intravenous lidocaine significantly contributes to the hemodynamic stability of adult patients undergoing general anaesthesia without having any further effect on the neuromuscular blockade.
Sane et al. [9]	2020	Original article	In patients undergoing lumbar laminectomy, wound infiltration with ropivacaine and MgSO4, as opposed to bupivacaine and MgSO4, resulted in improved postoperative analgesia and considerably decreased postoperative opioid usage
Shen et al. [20]	2021	Original article	While the muscle relaxant qualities of MgSO4 might help relax muscles during surgery, taking too much of the medication can cause muscular weakness, which may damage respiratory muscles and increase the risk of respiratory problems
Magana et al. [8]	2021	Research article	However, our findings suggest that prophylactic MgSO4 treatment is linked with a more favourable hemodynamic response. Prophylactic administration of MgSO4 and lidocaine was successful in attenuating hemodynamic responses to the stress impact of laryngoscopy and intubation.
Kanamori et al. [12]	2023	Research article	It may be necessary to have an adequately high amount of ionized magnesium in order for magnesium to have a neurological impact.
Amer et al. [21]	2015	Research article	During paediatric general anaesthesia, magnesium resulted in considerably lower bi spectral values, less time to attain bi spectral values below 60, lower tidal volume, and lower respiratory rate.
Mendonça et al. [22]	2017	Scientific article	Research found that the hemodynamic response to tracheal intubation might be attenuated with lower dosages of MgSO4.
Elcano et al. [23]	2006	Randomized controlled trial	The effects of MgSO4 were decreased blood loss, heart rate, arterial pressure, and surgical time. Moreover, the needs for aesthetic dosage and emergence time are modified by magnesium infusion.
Lee and Kwon et al. [24]	2009	Original article	During the pre-delivery phase, bispectrality and arterial pressure rises were decreased by preoperative MgSO4.

MgSO4 and muscle relaxation

Due to its proven ability to induce and sustain muscular relaxation, MgSO4 is a valuable adjuvant in various medical situations, such as anesthesia and disorders of the muscles [25]. Magnesium ions competitively obstruct calcium channels at the neuromuscular junction [26]. Magnesium inhibits acetylcholine release by interfering with calcium influx, which hinders muscular contraction. This process is essential to achieve and sustain muscular relaxation during anaesthesia-induced surgical operations [21,27]. Moreover, magnesium ions are antagonists of the N-methyl-D-aspartate (NMDA) receptor. When these receptors are triggered, excitatory neurotransmission is facilitated. Magnesium can lessen excitatory impulses by inhibiting NMDA receptors, which may result in muscular relaxation and analgesic benefits [28]. The neurotransmitter acetylcholine, which starts muscular contractions, can be inhibited by high magnesium levels. This inhibition encourages relaxation and adds to the general decrease in athletic activity. Because of its effects on vascular smooth muscle, MgSO4's vasodilatory qualities can enhance blood flow to muscles [29].

Analgesic and neuroprotective effects of MgSO4

Beyond its use as a muscle relaxant, MgSO4 has been studied in various medical contexts for its analgesic qualities. It employs many ways to modify the perception and transmission of pain. In the central nervous system, MgSO4 is an NMDA receptor antagonist. The action of NMDA receptors transmits pain signals. Magnesium may lessen pain perception and alter pain pathways by inhibiting these receptors. Magnesium competes with calcium ions and inhibits calcium channels, similar to how it works to relax muscles. The release of neurotransmitters involved in pain signaling may be impacted by this interference, which may lessen the severity of pain [22,30,31]. It has been proposed that magnesium possesses anti-inflammatory qualities, which may influence pain perception indirectly. Magnesium's anti-inflammatory properties may reduce pain when inflammation plays a role in pain. Because of the vasodilatory actions of magnesium, there is a possibility that reduced inflammatory mediators, improved circulation to the afflicted regions, and tissue repair will all contribute to decreased pain. Numerous neurotransmitters are regulated in part by magnesium ions. Changes in the amounts of neurotransmitters like substance P and glutamate can affect how pain is processed, which is one way that magnesium's analgesic effects work. Although MgSO4 shows potential as an analgesic, this substance has different therapeutic applications in treating pain. It has occasionally been used in conjunction with other analgesics to improve pain relief, especially in cases of neuropathic pain and postoperative pain [19,32,33].

The possible neuroprotective properties of MgSO4 have drawn attention, especially in brain damage or malfunction cases. There are several processes behind its capacity to shield nerve tissues and prevent harm. The NMDA receptors, essential for excitatory neurotransmission, are antagonistically affected by MgSO4. Overactivation of these receptors may result in excitotoxicity, which damages neurons. The capacity of magnesium to obstruct these receptors may shield neurons from overstimulating and ensuing damage. MgSO4 decreases calcium input into cells by blocking calcium channels. Since processes leading to cell death are linked to elevated intracellular calcium levels, magnesium helps preserve cellular homeostasis. It may even shield neurons from harm by preventing this calcium influx [34].

Increased blood flow might aid in the rehabilitation and health of neurons after damage. Magnesium affects glutamate release, one of the principal excitatory neurotransmitters. Glutamate dysregulation has been linked to neuronal damage; nevertheless, magnesium's capacity to control its release may offer protection against glutamate-mediated neurotoxicity [23,35].

Cardiovascular stability and reduction of aesthetic requirement

Because MgSO4 affects heart rhythm, vascular tone, and general hemodynamics, it can impact cardiovascular stability. As a vasodilator, MgSO4 causes peripheral vasodilation by relaxing vascular smooth muscle. This may have the effect of lowering blood pressure by reducing systemic vascular resistance. Nonetheless, there are circumstances in which preserving or reaching stable blood pressure may be advantageous due to the vasodilatory impact. Magnesium ions are essential to keep the heart's electrical activity regular. The antiarrhythmic qualities of MgSO4 may lower the risk of certain arrhythmias by stabilizing cell membranes, regulating cardiac conduction, and modifying ion channels [24,36].

Magnesium can affect the heart's conduction system and myocardial contractility by inhibiting calcium channels. This activity may aid its capacity to control heart rhythm and rate, which might result in cardiovascular stability. The possible preventive benefits of magnesium against ischemia have been examined. Magnesium's capacity to dilate blood vessels and maybe lower the danger of spasms may offer protection against ischemic damage when there is decreased blood flow to the heart or other organs. Because of its vasodilatory properties, MgSO4 can affect how blood pressure is regulated. However, the effect on blood pressure might change according to dosage, specific patient variables, and the situation [37,38].

Enhancing the effects of anesthetics may achieve the appropriate aesthetic depth at lower dosages of these medicines by reducing the release of acetylcholine and blocking calcium channels. As a result, when MgSO4 is given in addition to anesthesia for muscular relaxation, the amount of aesthetic may be reduced. There are some soothing effects from MgSO4 [39-41].

Because of its analgesic qualities, it may be possible to manage pain more effectively and need fewer additional analgesics or anesthetics to treat pain during or after surgery. This might make using these medications at lower dosages possible, reducing any adverse effects. Because of its vasodilatory properties and impact on heart rhythm, MgSO4 may help maintain cardiovascular stability when under anesthesia. A smoother anesthetic course may be possible with improved circulatory stability, maybe needing fewer modifications or extra medications to keep hemodynamic parameters within the intended range. Adjunctive use of MgSO4 may help reduce some of the adverse effects associated with larger dosages of anesthetics, such as respiratory depression, hemodynamic instability, or delayed recovery from anesthesia, by perhaps allowing for lower doses of primary anesthetics [42-44].

Complications associated with MgSO4 as an anesthetic agent

Although MgSO4 has certain advantages over conventional analgesics, such as opioids in general anesthesia, there are some drawbacks and difficulties with its usage. Respiratory depression may result from higher dosages of MgSO4, mainly if additional anesthetics are being used at the same time. Hypoxia and respiratory impairment may ensue from this decreased breathing rate and depth [45]. Because of its vasodilatory effects, MgSO4 may be troublesome for individuals with reduced cardiovascular function since it might produce hypotension or decrease blood pressure. Excessive vasodilation can cause circulatory collapse in severe situations. While the muscle relaxant qualities of MgSO4 might help relax muscles during surgery, taking too much of the medication can cause muscular weakness, which may damage respiratory muscles and increase the risk of respiratory problems [46].

MgSO4 may impact cardiac conduction, resulting in bradycardia or other irregularities in cardiac rhythm. People who already have heart problems may experience this impact more strongly. MgSO4 may intensify the effects of several neuromuscular blocking drugs used in anesthesia, resulting in extended neuromuscular blockade or muscle weakening. Overdosing on MgSO4 can result in depression of the central nervous system, which can induce sleepiness, disorientation, or, in extreme situations, coma. Although uncommon, allergic reactions to MgSO4 can happen and present as itching, skin rashes, or more systemic severe responses. Extended prolonged overdosing on MgSO4 has the potential to cause electrolyte abnormalities, such as hypermagnesemia, which can have a variety of systemic consequences [1].

Research has looked into the possibility of using MgSO4 to decrease the emergence of agitation in kids under general anesthesia. Emergence agitation is common, especially in pediatric patients, characterized by restlessness or disorientation emerging from anesthesia. Pediatric patients' emergence agitation ratings may decrease if MgSO4 is administered as part of the anesthesia. Less agitation, fewer mood swings episodes, and better postoperative conduct might all be signs of this agitation. Because of its moderate sedative qualities, MgSO4 may help facilitate a faster recovery from anesthesia. It could have anxiolytic effects, which could lessen the tension and worry that comes with waking up from anesthesia. However, a study suggests that While administering MgSO4 to pediatric patients under general anesthesia lengthens their recovery period without causing any other adverse effects, it has no discernible influence on the incidence of emergence agitation in these individuals [9,20].

Due to their possible synergistic effects, MgSO4 and lidocaine have been investigated in a variety of medical scenarios. Lidocaine reduces nerve transmission and produces analgesia by blocking sodium channels. Although MgSO4 is not a local anesthetic, it does have analgesic qualities and may intensify the effects of lidocaine in some situations, such as pain management treatments or regional anesthesia. When combined with lidocaine, MgSO4 may enhance its analgesic effects by altering the routes via which pain is transmitted or enhancing the effects of local anesthetics. This combination may result in less lidocaine being used and better pain management. Even in the presence of MgSO4, intravenous lidocaine significantly contributes to the hemodynamic stability of adult patients undergoing general anesthesia without having any further effect on the neuromuscular blockade. MgSO4 improves the train of four recovery rates without T1 recovery and extends the NMB recovery features without changing the onset speed [19].

By reducing the need for opioid-based analgesics, this combination may minimize the mother's exposure to opioid-related adverse effects. MgSO4 and clonidine have been looked into in specific research as anesthetic adjuncts during C-sections. They are used to regular anesthetics to potentially lower total anesthetic needs and enhance postoperative pain management. By lowering the number of main anesthetic agents needed, using MgSO4 and clonidine in addition to anesthetics during a C-section may help minimize some anesthetic side effects, such as nausea or hypotension. Research indicates that combining clonidine and MgSO4 may work synergistically to provide analgesia and enhance the mother's overall perioperative experience. During pregnancy, general anesthesia is a complicated process. MgSO4 or clonidine infused intravenously would maintain hemodynamic parameters and deepen general anesthesia in the context of surgical delivery, posing no additional acute danger to newborns [18,47].

Complications associated with MgSO4

Although it is usually regarded as safe when used under proper medical care, using it as an adjuvant to anesthesia may have some risks. MgSO4 can cause respiratory depression at larger dosages, which results in a reduction in breathing depth and rate. Those who have impaired respiratory function may find this to be extremely dangerous. Vasodilation brought on by MgSO4 can lower blood pressure. Patients with pre-existing cardiovascular issues or those sensitive to changes in blood pressure may find this impact bothersome [29]. Excessive MgSO4 dosages might impact respiratory muscles and could result in breathing issues by causing muscular weakening or paralysis. Overdosing on magnesium can cause central nervous system depression, which can manifest as sleepiness, disorientation, or, in extreme situations, coma. Although MgSO4 is frequently used to treat arrhythmias, taking too much of it might cause heart block, bradycardia, or cardiac depression. The interaction of MgSO4 can heighten the effects of other anesthetic drugs. When used with other anesthetic medications, this may cause unanticipated problems or adverse consequences. Although uncommon, allergic reactions to MgSO4 can occur in certain people and cause symptoms, including rash, irritation, or anaphylaxis. Renal impairment may result from long-term usage or high dosages of MgSO4 that affect kidney function in sensitive individuals [48].

Prenatal MgSO4 has been linked to maternal adverse effects that are commonly observed. While severe life-threatening occurrences in obstetrics are rare, they can occur and include cardiac arrest, respiratory arrest, altered heart function, and even death. Flushing, headaches, perspiration and fever, nausea, vomiting, impaired vision, and soreness at the injection site or intramuscular site are a few of the less typical minor adverse effects. A greater frequency of mild side effects, such as flushing, tachycardia, hypotension, nausea, vomiting, sweating, and injection site issues [49-51].

Multiple recent papers have proven the effectiveness and safety of antenatal MgSO4 (4g loading dose, 1-g/h maintenance) in avoiding cerebral palsy. Currently, the most preferred, straightforward, and affordable approach to employ daily may be to administer just 4 grams of MgSO4 in cases of strongly suspected early preterm birth, followed by 4 grams in cases of unavoidable preterm birth. It is seen as effective without sacrificing the element of safety. It should be mentioned that studies on additional neuroprotective substances, such as melatonin, are now underway [52-54].

Although an established medication, MgSO4 possesses many properties that are highly advantageous for anesthesiologists. When administered judiciously to augment analgesia and muscle relaxation in surgical patients, its utilization can significantly enhance the overall outcome of surgical procedures. In addition to its long-standing presence in medical practice, MgSO4 exhibits a range of pharmacological attributes that render it particularly beneficial in anesthesiology. Its ability to potentiate analgesia and promote muscle relaxation is a crucial pillar in managing surgical patients undergoing various procedures. By incorporating MgSO4 into anesthesia protocols with careful consideration and precise dosing, anesthesiologists can harness its therapeutic potential to optimize perioperative care and contribute to favorable surgical outcomes. The standard protocol for administering MgSO4 involved a loading dose of 30-50 mg/kg and a maintenance dose of 6-20 mg/kg/h (constant infusion) until the procedure was completed.

Nonetheless, in several earlier studies, postoperative analgesia was also successfully treated with a single magnesium bolus without a continuous infusion. An infusion of MgSO4 has been shown to lessen postoperative pain. One study assessed the impact of MgSO4 administered before surgery on obstetric patients' postoperative discomfort [55,56]. They found that giving patients an intravenous MgSO4 bolus before putting them under general anesthesia can lessen the impact of morphine consumption on postoperative VAS scores and the first 24 hours after surgery. Likewise, our Mg groups' VAS scores were significantly lower during the study period.

On the other hand, a different study looked at the impact of intravenous magnesium infusions on pain following complete knee arthroplasties performed under spinal anesthesia. Following intravenous MgSO4 infusions, they observed no appreciable changes in the patients' levels of postoperative pain or painkiller intake. One method of managing postoperative pain in pregnant women is to administer a bolus of 50 mg/kg MgSO4 before inducing general anesthesia. This can significantly reduce the amount of morphine needed during the initial post-operative phase.

## Conclusions

Because high dosages of MgSO4 might cause problems, careful monitoring and prudent management are necessary while administering this medication. More research is needed to determine the best ways to lose it, understand how it works, and pinpoint the patient demographics who might most benefit from its supportive function in general anesthesia. In gravid patients, a small amount of MGSO4 can effectively alleviate the pain in the post-labor scenario. Because of its many uses, MgSO4 offers a route for more advanced aesthetic procedures that might improve patient safety and recovery in surgical settings. It is more successful as a coadjutant in many instances, from pediatrics to geriatric. MgSO4 can have adverse effects such as hypotension, respiratory depression, and neuromuscular blockade, especially at higher doses, even though it is generally regarded as safe when used at the recommended dosages and under medical care.
